# Metabolomics analysis of visceral leishmaniasis based on urine of golden hamsters

**DOI:** 10.1186/s13071-023-05881-3

**Published:** 2023-08-30

**Authors:** Dongmei Yuan, Jianping Chen, Zhiwei Zhao, Hanxiao Qin

**Affiliations:** 1 Department of Human Anatomy, West China School of Basic Medical Sciences and Forensic Medicine, Sichuan University, Chengdu, 610041 Sichuan People’s Republic of China; 2 Department of Pathogenic Biology, West China School of Basic Medical Sciences and Forensic Medicine, Sichuan University, Chengdu, 610041 Sichuan People’s Republic of China; 3 Clinical Trial Center, Chengdu Second People’s Hospital, Chengdu, 610021 Sichuan People’s Republic of China

**Keywords:** *Leishmania*, Visceral leishmaniasis, Metabolomics, Biomarker panel

## Abstract

**Background:**

Leishmaniasis is one of the most neglected tropical diseases and is spread mainly in impoverished regions of the world. Although many studies have focused on the host’s response to *Leishmania* invasion, relatively less is known about the complex processes at the metabolic level, especially the metabolic alterations in the infected hosts.

**Methods:**

In this study, we conducted metabolomics analysis on the urine of golden hamsters in the presence or absence of visceral leishmaniasis (VL) using the ultra-performance liquid chromatography (UPLC) system tandem high-resolution mass spectrometer (HRMS). The metabolic characteristics of urine samples, along with the histopathological change and the parasite burden of liver and spleen tissues, were detected at 4 and 12 weeks post infection (WPI), respectively.

**Results:**

Amino acid metabolism was extensively affected at both stages of VL progression. Meanwhile, there were also distinct metabolic features at different stages. At 4 WPI, the significantly affected metabolic pathways involved alanine, aspartate and glutamate metabolism, the pentose phosphate pathway (PPP), histidine metabolism, tryptophan metabolism and tyrosine metabolism. At 12 WPI, the markedly enriched metabolic pathways were almost concentrated on amino acid metabolism, including tyrosine metabolism, taurine and hypotaurine metabolism and tryptophan metabolism. The dysregulated metabolites and metabolic pathways at 12 WPI were obviously less than those at 4 WPI. In addition, seven metabolites that were dysregulated at both stages through partial least squares-discriminant analysis (PLS-DA) and receiver-operating characteristic (ROC) tests were screened to be of diagnostic potential. The combination of these metabolites as a potential biomarker panel showed satisfactory performance in distinguishing infection groups from control groups as well as among different stages of infection.

**Conclusion:**

Our findings could provide valuable information for further understanding of the host response to *Leishmania* infection from the aspect of the urine metabolome. The proposed urine biomarker panel could help in the development of a novel approach for the diagnosis and prognosis of VL.

**Graphical Abstract:**

**Figure:**
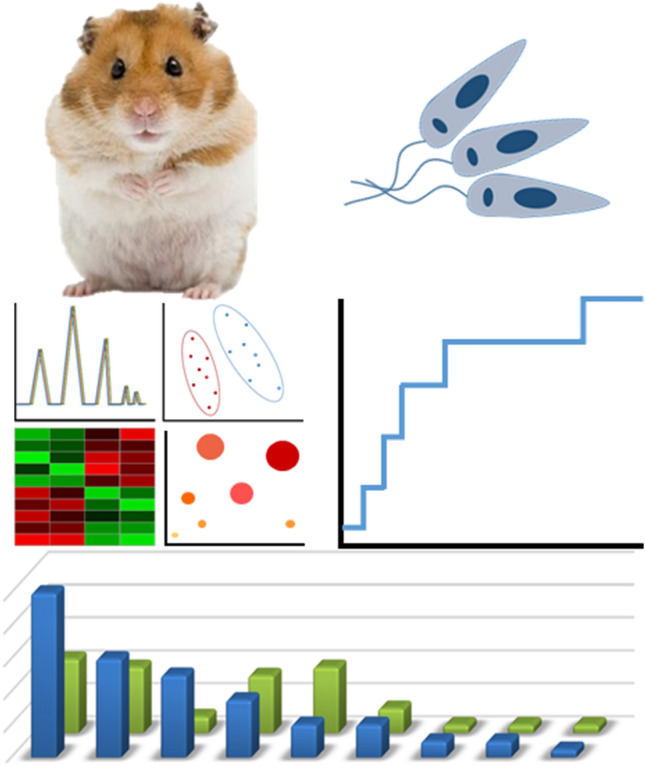

**Supplementary Information:**

The online version contains supplementary material available at 10.1186/s13071-023-05881-3.

## Background

Caused by over 20 parasite species of the genus *Leishmania*, sandfly-borne parasitosis leishmaniasis, one of the most neglected tropical diseases, is spread in approximately 97 countries globally [1]. An estimated 0.7–1 million new cases of leishmaniasis per year are reported [2]. The manifestations of leishmaniasis mainly include VL, cutaneous leishmaniasis (CL) and mucocutaneous leishmaniasis (MCL). VL is the most severe form and can be fatal if not treated in time. In recent years, VL cases were concentrated in East Africa, the Indian subcontinent and Brazil, and 83% of these cases were reported in Brazil, Ethiopia, India, South Sudan and Sudan [1, 3]. In China, it has been acknowledged that VL is the only autochthonous form of leishmaniasis, mainly distributed sporadically in local areas of Xinjiang, Gansu and Sichuan provinces, and the endemic area is gradually expanding [4]. Through the previous discrimination and phylogenetic analysis, *Leishmania donovani* and *L. infantum* have been confirmed as the major causative agents of Chinese VL [5, 6].

There is no doubt that early diagnosis is crucial for the epidemiological investigation and control of leishmaniasis, as well as for its treatment. Additionally, considering the very diverse reservoir hosts in natural epidemic focuses and the complex mobility of the population, an ideal detection/diagnosis method should be simple and rapid and suitable for high-throughput screening. Laboratory examination methods of VL have been studied, including serological tests, immunological assays and PCR-based methods. Some of them, such as the rK39 dipstick, have been commercially applied. Due to the sensitivity and specificity, there is no other method now that can make final diagnosis of VL than invasive biopsies of spleen, liver, bone marrow and lymph nodes. In particular, splenic aspiration is still the current gold standard [7]. These life-threatening invasive methods require relatively high operation skills and hence are not suitable for large-scale popularization or field investigation [8]. Therefore, the development of a new rapid and effective diagnostic method is still necessary. For treatment, pentavalent antimonial (SbV), amphotericin B and miltefosine have long been used for VL therapy. The ever-rising challenges of drug resistance, adverse reactions and toxicity have undermined the effectiveness of chemotherapy for VL [9]. There is an urgent need for the development of new specific medicine to improve the current situation. In summary, improvements in either the diagnosis or pharmacotherapy of VL rely essentially on comprehensive knowledge of the mechanisms that underlie leishmaniasis.

With extensive studies, more comprehensive knowledge has been obtained about the molecular mechanisms of leishmaniasis pathogenesis [10, 11]. In recent years, some discoveries have been made in the molecular metabolism of *Leishmania* with the introduction of metabolomics approaches. The metabolic characteristic of different stages or different species of *Leishmania* has been described [12–14], and the metabolomic profile of host cells has been found to be affected during the *Leishmania* infection [15–17]. Additionally, deviation in the metabolome induced by drugs (SbV or miltefosine) has been reported [18, 19], as well as the metabolomic analyses of the mechanism of drug resistance in *Leishmania* [20–22]. These studies have broadened our current knowledge of the host response to *Leishmania* invasion. On the other hand, through comparative statistical analyses on acquired datasets, mass spectrometry-based metabolomics methods have unique advantages in searching indicators for certain statuses, which has been well demonstrated and applied by studies searching for biomarkers of a variety of diseases [23]. For leishmaniasis, some metabolites that were predictive and prognostic for the treatment of leishmaniasis have been selected and evaluated [24]. In addition, our recent study explored the metabolic changes in serum over a four time point series of VL. Metabolic pathways in connection to glycerophospholipids (GPLs) were affected significantly, in which phosphatidylcholine (PC) and phosphatidylethanolamine (PE) were upregulated during VL [25]. Overall, by directly investigating changes in small molecules during infection, metabolomics approaches provide a promising means for better understanding host-*Leishmania* interplay and discovering novel targets for the diagnosis and prognosis of leishmaniasis. Nevertheless, the current understanding of host-*Leishmania* interaction from metabolic aspects is still insufficient [26].

Since urine is the sample type that contains most metabolic end-products, changes in urine have the ability to reflect the disturbance of many body biochemical pathways [27, 28]. Additionally, because of its easier availability and less complexity in components than other biospecimens, urine is an ideal type of sample for studies and tests [29]. In the research field of *Leishmania*, the Syrian golden hamster (*Mesocricetus auratus*) is highly susceptible to infection and can simulate the progression of VL well; hence, it is deemed the best experimental model for pathological study [30]. In this study, we conducted metabolomics analysis on the urine of golden hamsters in the presence or absence of VL based on the ultra-performance liquid chromatography (UPLC) system tandem high-resolution mass spectrometer (HRMS), and univariate and multivariate statistical methods were used to dissect the dataset acquired from both the early and late stages of VL. The findings on the characteristics of the metabolome could facilitate our understanding of the host response to VL progression. Moreover, the selected potential biomarker is expected to contribute to the development of novel diagnostic techniques and process monitoring of VL.

## Methods

### Ethical statement

The animal experiments in this study were permitted by and under supervision of the Medical Ethics Committee of Medical Management Department, Sichuan University. In case of operations that might cause pain in animals, ether inhalation anaesthesia was given prior to other processes. The Ethics Certification for Animal experiments and its translated version are provided in the Supplementary Materials.

### *Leishmania* strain and infection model

The *Leishmania* strain used in this study was identified as *L. infantum* in a previous study [25]. The strain was routinely conserved in hamsters. Before experiments, the *Leishmania* strain was isolated and cultured in modified M199 liquid medium (containing 15% foetal bovine serum and 20% sterile defibrinated rabbit blood) hermetically at 26 °C with shaking at 80 rpm. Metacyclic promastigotes were concentrated and resuspended in pH 7.4 phosphate-buffered saline (PBS) prepared for animal infection.

A total of 24 2-month-old female golden hamsters (*M. auratus*) were divided randomly into control and infection groups (*n* = 12 for each group). All hamsters were bred in a laboratory animal room and provided sterile food and water ad libitum. Before formal experiments, 4 weeks of breeding was given to attenuate stress responses.

Similar to our previously established model, the grouped hamsters were subjected to inoculation. After counting, 1 ml of PBS inoculum containing 2.7 × 10^7^ *L. infantum* promastigotes was intraperitoneally injected into each hamster of the infection group [25]. For the control group, the hamsters were subjected separately with 1 ml PBS only.

### Sample collection and evaluation of the infection model

At both 4 and 12 WPI, eight urine samples in each group were collected for metabolomics detection. To eliminate errors caused by the circadian rhythm of mammals [31], sampling was started at the same time of the day, performed over 6 h through metabolism cages. Before collection, overnight fasting and adequate drinking water were given. The urine samples were immediately stored at –80 °C until metabolomics detection.

In each group, the other four hamsters were killed for infection confirmation and pathological observation. Two hamsters from the infection or control group were killed at 4 and 12 WPI, respectively. The liver and spleen tissues were collected and fixed in 10% paraform (Solarbio, Beijing, China) for 4 weeks, followed by a procedure of gradually substituting ethanol for water in fixed tissues. Then, xylene was gradually substituted with ethanol, and the sections were finally adequately saturated with liquid paraffin. Finally, 3 μm tissue slices were dyed with haematoxylin-eosin (H&E) reagent (Solarbio, Beijing, China). The progression of infection was evaluated through microscopic examination.

In parallel, the liver and spleen tissues were weighed, and 50 mg of each sample was homogenized in 1 ml PBS. Then, the total DNA was extracted using the TIANamp Genomic DNA Kit (TianGen Biotech, Beijing, China) per the manual. For each sample, the extracted DNA was diluted in 80 μl RNase-free ddH_2_O. For the standard curve of *Leishmania* load, the parasites were diluted in a tenfold gradient from 10^6^ to 1. Total DNA was extracted and stored in 80 μl RNase-free ddH_2_O. A TaqMan probe fluorescence real-time PCR scheme was conducted to detect parasite load. The primer pair and probe were described in previous research [32]. The PCR conditions are listed in Additional file 1: Table S1. PCR programs were run on Mastercycler ep realplex 2 (Eppendorf, Germany).

### Protocol of metabolomics detection

The metabolomics detection of urine samples was conducted at the laboratory of The Beijing Genomics Institute (BGI) based on the platform of the Waters 2D Ultra Performance Liquid Chromatography (UPLC) system tandem ThermoFisher Q Exactive High Resolution Mass Spectrometer (HRMS).

The detailed process of sample pretreatment and the component of inner standards are provided in Additional file 2. Chromatographic separation was performed on a BEH C18 column (1.7 μm, 2.1 × 100 mm, Waters, USA). The mobile phase of positive electrospray ionization (ESI+) mode consisted of solution A (water containing 0.1% formic acid) and solution B (methanol containing 0.1% formic acid). The mobile phase of negative electrospray ionization (ESI−) mode consisted of solution A (water containing 10 mM ammonium formate) and solution B (95% ethanol containing 10 mM ammonium formate). The gradient elution procedure was as follows: 0–1 min, 2% solution B; 1–9 min, 2–98% solution B; 9–12 min, 98% solution B; 12–12.1 min, 98–2% solution B; 12.1–15 min, 2% solution B. The flow velocity was 0.35 ml/min, the column temperature was 45 °C, and the sample loading volume was 5 μl.

The MS and MS_2_ data were obtained with scanning mass/nucleus ratios ranging from 70 to 1050. The injection times of primary and secondary scanning were 100 and 50 ms, respectively. The stepped NCE was 20, 40 and 60 eV. The sheath gas flow rate of ESI was 40, and the aux gas flow rate was 10. The spray voltage was 3.80 for ESI+ and 3.20 for ESI−. The capillary temperature was 320 °C, and the auxiliary gas heater temperature was 350 °C.

### Statistical analyses and data mining

The raw data were primarily imported into Compound Discoverer 3.1 (Thermo Fisher Scientific, USA) for preprocessing. Peak picking, alignment and extraction, retention time correction, adduct ion merger, missing value filling, background peak marking, normalization, deconvolution and identification were carried out. After preprocessing, original data were transferred into the R-based software package metaX [33] to perform metabolomics analyses, statistical analyses and metabolite annotations. After probabilistic quotient normalization (PQN) and quality control-based robust LOESS signal correction (QC-RLSC), ion peaks with a coefficient of variation (CV) in QC samples > 30% were eliminated from downstream analyses. Then, the detected ions were identified through comparison with standard substances and by combining references of self-built databases, commercial databases and open-access libraries, including the BGI Library, mzCloud Mass Spectral Library, Chemspider, Human Metabolome Database (HMDB), Kyoto Encyclopedia of Genes and Genomes (KEGG) and Lipidmaps.

After ion identification, pretreated data were subjected to standard statistical analyses. Before univariate and multivariate statistical analyses, original data were normalized through log2 conversion and Pareto scaling. Principal component analysis (PCA) was then employed to observe similarities and dissimilarities within and between sample groups and to evaluate the outliers. Student's t-test, fold change (FC) computation and PLS-DA were conducted to identify differential ions between the infection and control groups. By computing FC values, false discovery rate (FDR) adjusted *P*-values and the variable importance for the projection (VIP), metabolites satisfying FC ≥ 1.2 or ≤ 0.8333, *P* < 0.05 and VIP ≥ 1.0 simultaneously were recognized as differential metabolites.

To maximize the accuracy of the results, only ions whose MS_2_ data were fully matched to libraries were selected and used in the analyses of this study. Metabolites were classified per their category information on HMDB. The pathway analyses were conducted using MetaboAnalyst 5.0 [34]. The performance of potential biomarkers for diagnosing visceral leishmaniasis was evaluated through the ROC test. The closer the area under the curve (AUC) was to 1.0, the better the effectiveness of the predictive diagnosis.

## Results

### Behaviour of animals and progression of infection

After infection, there were no significant differences in the behaviour of hamsters between the control and infection groups at 4 WPI. At 12 WPI, hypersomnia and reduced activity were observed in the hamsters of the infection group.

**Fig. 1:**
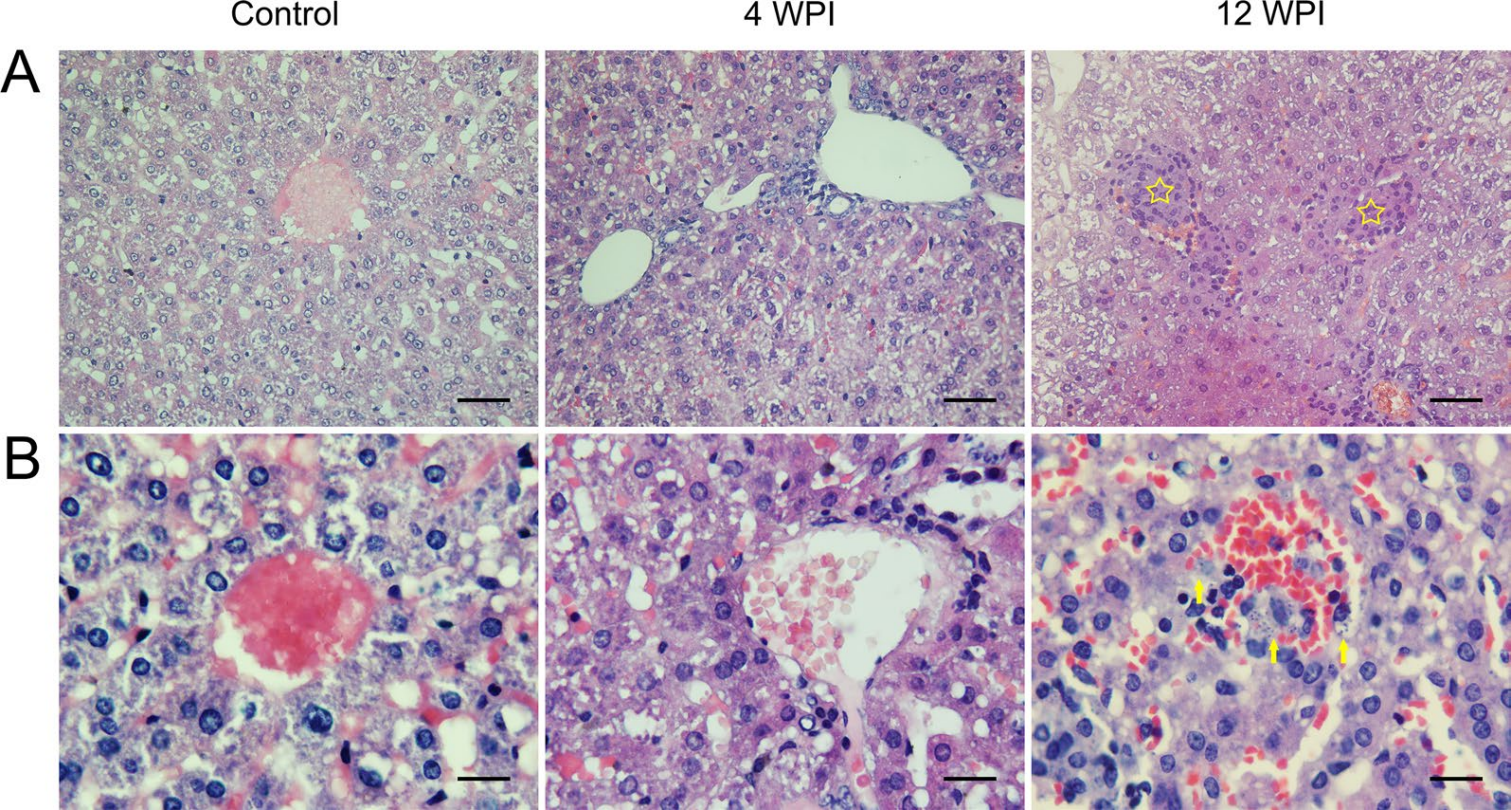
H&E-dyed pathological slices of liver tissues. **A** Observed tissue sections at 400 × magnification . **B** Observed tissue sections at 1000 × magnification. Graphs in each column from left to right represent groups of control, 4 and 12 WPI, respectively, as marked on the top. In contrast to the control group, the infiltration of inflammatory cells was observed at 4 WPI. Significant sporadic granulomas were observed at 12 WPI, denoted by yellow pentagons. *Leishmania* amastigotes were denoted by yellow arrows. Bar: (**A**) 60 μm; (**B**) 24 μm

**Fig. 2:**
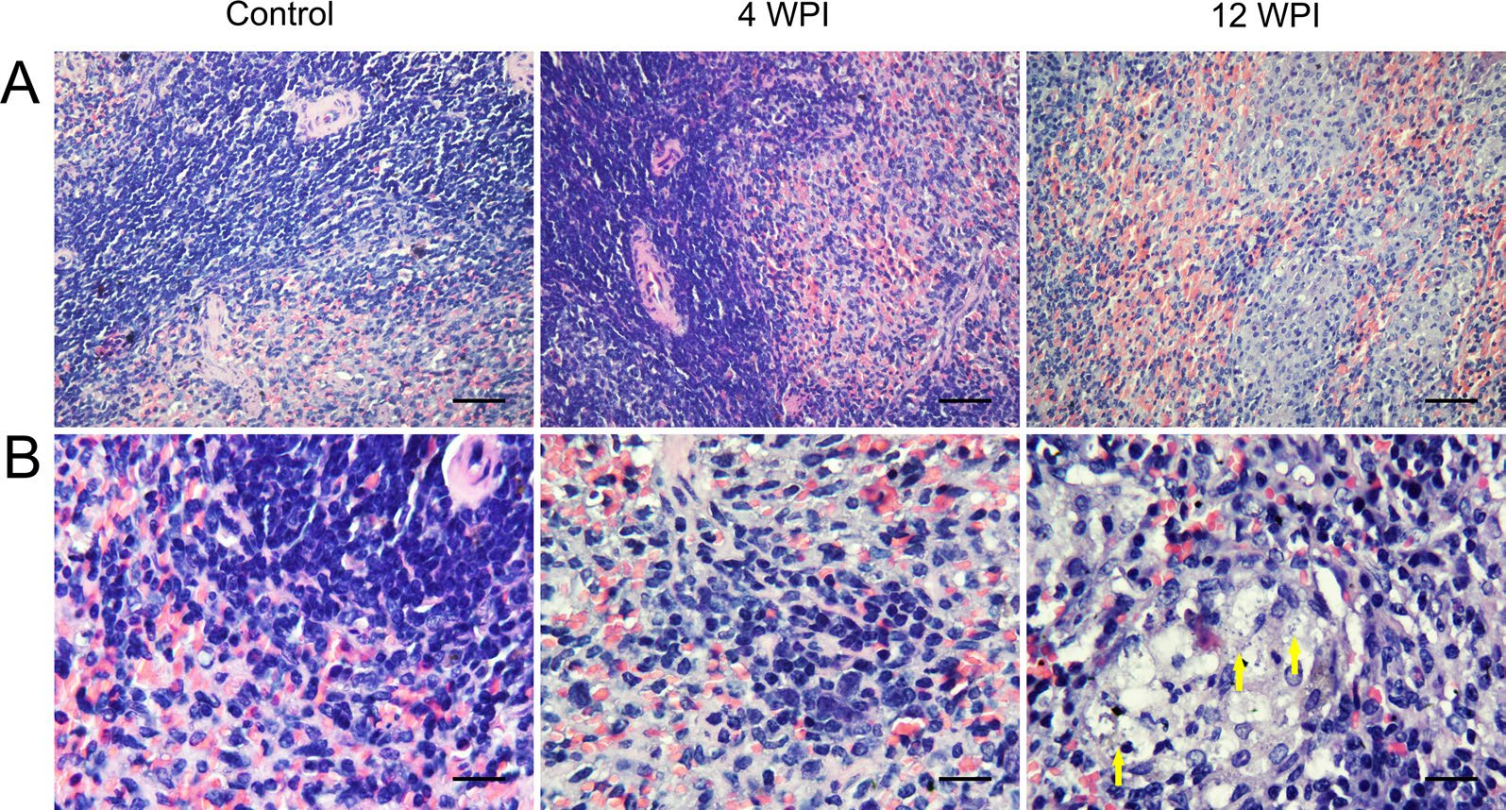
H&E-dyed pathological slices of spleen tissues. **A** Spleen tissue sections at 400 × magnification. **B** Spleen tissue sections at 1000 × magnification. Graphs in each column from left to right represent groups of control, 4 and 12 WPI, respectively, as marked on the top of the graphs. In contrast to the control group, the changes were not as obvious, yet a trend of macrophage aggregation was recognized. *Leishmania* amastigote-like spots were obvious in expanded white pulp at 12 WPI, denoted by yellow arrows. Bar: (**A**) 60 μm; (**B**) 24 μm

**Fig. 3:**
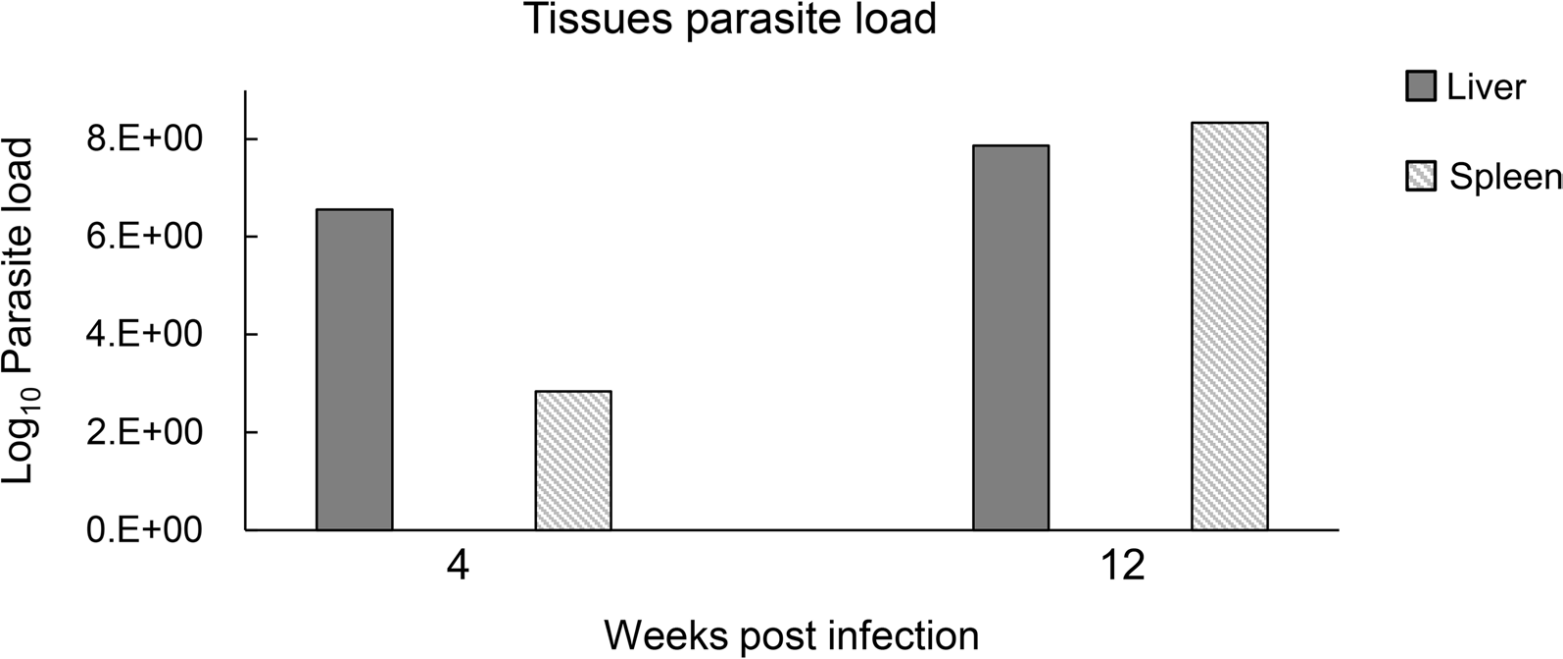
Parasite load in the liver and spleen. *Leishmania* load in liver and spleen at 4 and 12 WPI, respectively (2 hamsters from the infection or control group were killed at each time point and each sample was in triplicate)

Microscopy of the tissue slices revealed the progression of VL in the infection group compared with the control group (Figs. 1, 2). At 4 WPI, obvious infiltration of inflammatory cells was observed in liver slices, while the changes in spleen were inconspicuous. When VL progressed to 12 WPI, sporadic granulomas began to appear in the liver slices. In the spleen, expanded white pulp was observed, within which diffuse amastigotes were found. The infection status was also determined by real-time PCR (real-time PCR standard curve is shown in Additional file 3: Fig. S1), in which the parasite load showed an increasing trend from 4 to 12 WPI (Fig. 3), which is generally consistent with the pathological results. These results were also consistent with our previous study [25], which proved that the pathological features of the VL animal model were stable and repetitive.

### Overview of metabolomics detection

The base peak chromatograms (BPCs) of the QC samples were highly overlapped, and the PCA plots of the QC samples were tightly concentrated. These results demonstrated the repeatability and stability of the LC-MS system over the testing period (Additional file 4: Fig. S2).

**Fig. 4:**
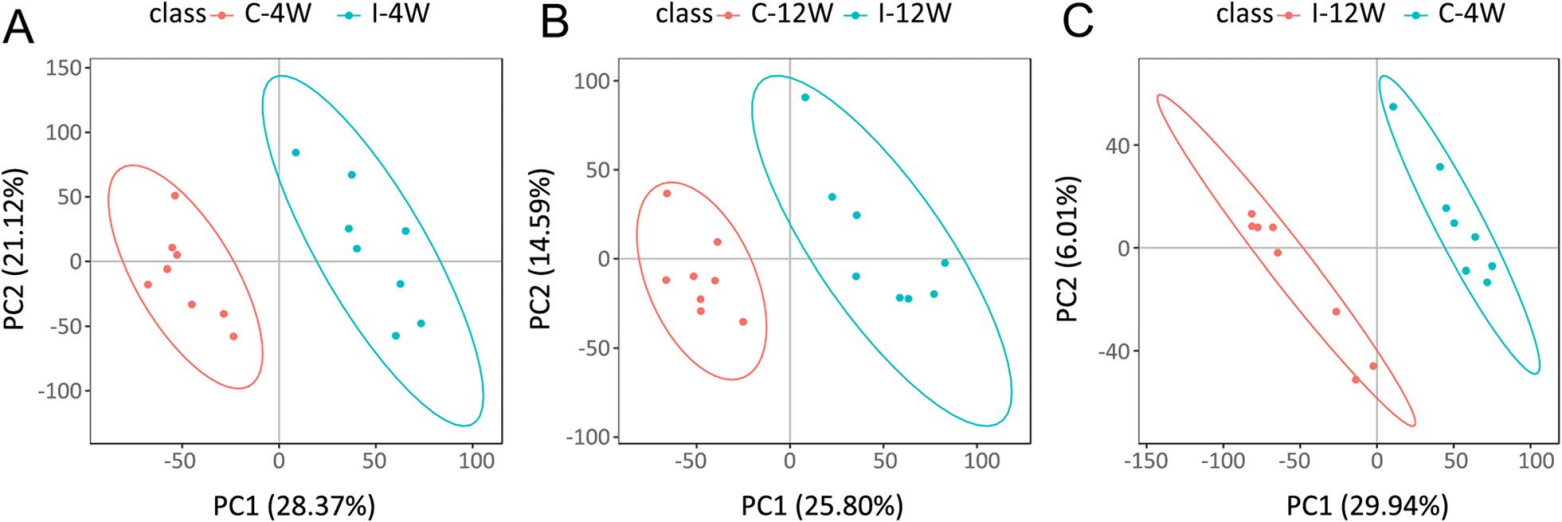
PLS-DA plots of comparison between different groups in ESI + mode. In the PLS-DA plot, each data point represents one urine sample. The results were computed through R-based package metaX. **A** Control vs. infection at 4 WPI. *R*2 = 0.99, *Q*2 = 0.82. **B** Control vs. infection at 12 WPI. *R*2 = 0.99, *Q*2 = 0.58. **C** 4 WPI vs. 12 WPI of the infection group. *R*2 = 0.99, *Q*2 = 0.60

At 4 or 12 WPI, eight urine samples from the infection or control group, respectively, underwent metabolite analysis. After raw data pre-processing, data filtration and metabolite annotation, a total of 18,201 ions were identified, of which 11,038 were in ESI+ mode and 7163 were in ESI− mode (Table 1A). These ions covered the identification confidence levels of level 1 to 5 [35]. In this study, to guarantee the accuracy and preciseness of the results, only the metabolites that were interpreted through MS_2_ libraries were selected for metabolomics analyses and interpretations. Since PCA can discriminate neither the infection group from the control group nor the infection group at different time points (Additional file 4: Fig. S2B), supervised PLS-DA was then employed to further identify the different metabolites among different groups (Fig. 4 in ESI+ mode and Additional file 5: Fig. S3 in ESI− mode). Figure 4A, B shows that the infection groups were significantly separated from the control groups at 4 WPI and 12 WPI, and the infection groups from 4 and 12 WPI were also divided in Fig. 4C. For Fig. 4A, *R*2 = 0.99, *Q*2 = 0.82; for Fig. 4B, *R*2 = 0.99, *Q*2 = 0.58; for Fig. 4C, *R*2 = 0.99, *Q*2 = 0.60.

**Table 1:** Distribution numbers of identified metabolites

(A) Numbers of metabolites of different confidence levels					
Detection mode	Total ions	Ions with RSD ≤ 30%	Roughly identified^a^	MS interpreted^a^	MS2 interpreted^a^
ESI+	11,524	11,038	4886	5511	641
ESI−	7463	7163	3188	3632	343

(B) Numbers of differential metabolites at different time points (FC ≥ 1.2 or ≤ 0.8333, *p* < 0.05 and VIP ≥ 1.0)			
Detection mode	Time points		Total
	4 WPI	12 WPI	
ESI+	57	47	104
ESI−	33	18	51
Total	90	65	155

^a^Confidence level of metabolites is in accord with the Metabolomics Standards Initiative (MSI) [35]. Roughly identified: metabolites of level 5; MS interpreted: the metabolites of level 4; MS2 interpreted: the metabolites of level 1–3

**Fig. 5:**
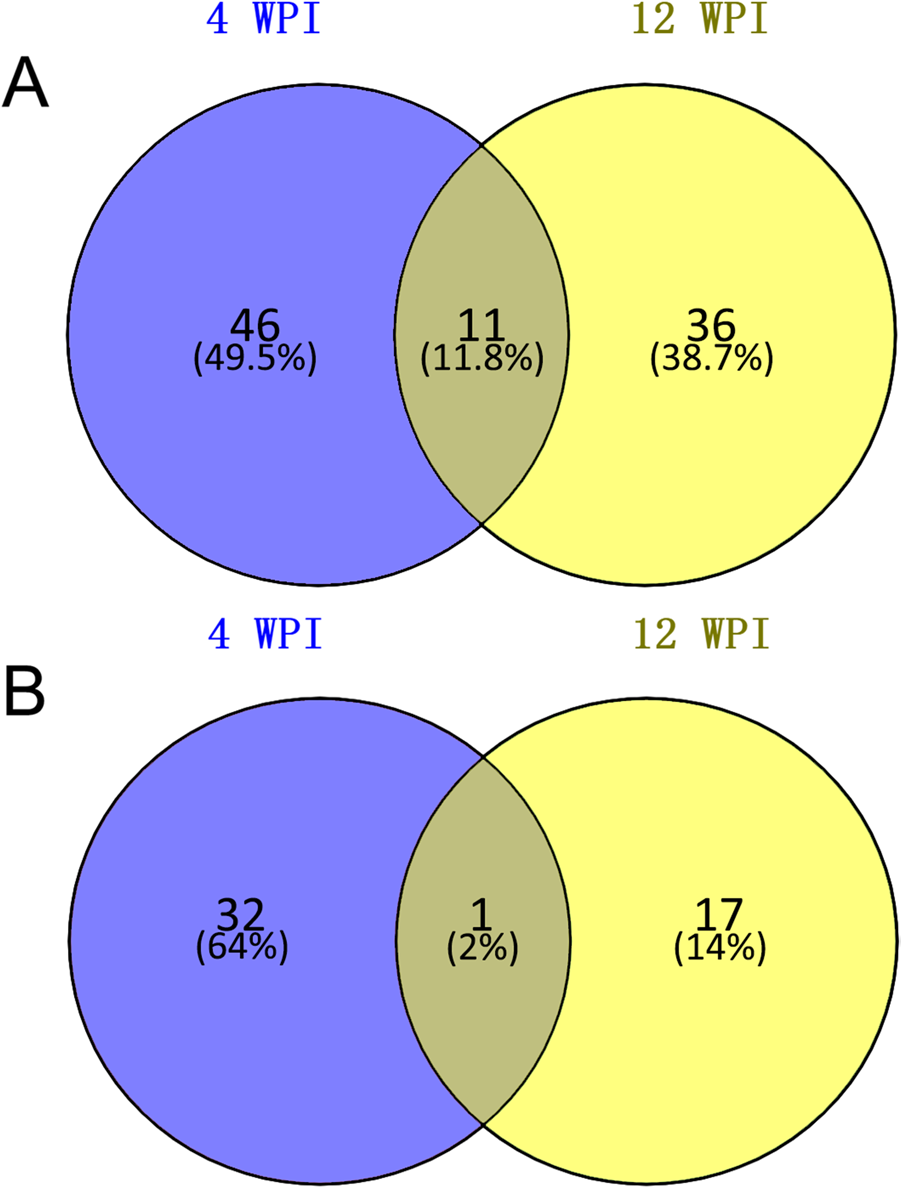
Count and distribution of differential metabolites at 4 WPI and 12 WPI. The total differential metabolites at different time points were visualized as Venn diagram based online “Venny 2.1”. **A** Results in ESI + mode. Eleven metabolites were shared at 4 WPI and 12 WPI. **B** Results in ESI− mode. One metabolite was shared at 4 WPI and 12 WPI

As Table 1B shows, following the preset criteria of FC ≥ 1.2 or ≤ 0.8333, FDR adjusted *p* < 0.05 and VIP ≥ 1.0, at 4 WPI, there were 90 differential metabolites between the infection and control groups, of which 57 were in ESI + mode and 33 were in ESI− mode. At 12 WPI, 47 differential metabolites in ESI + mode and 18 differential metabolites in ESI− mode were identified. The detailed information of the total differential metabolites is listed in Additional file 6. Notably, 12 metabolites varied at both 4 and 12 WPI (Fig. 5A, B), of which 11 were in ESI + mode and the other in ESI− mode.

### Characterization of the metabolome between the infection and control groups

**Fig. 6:**
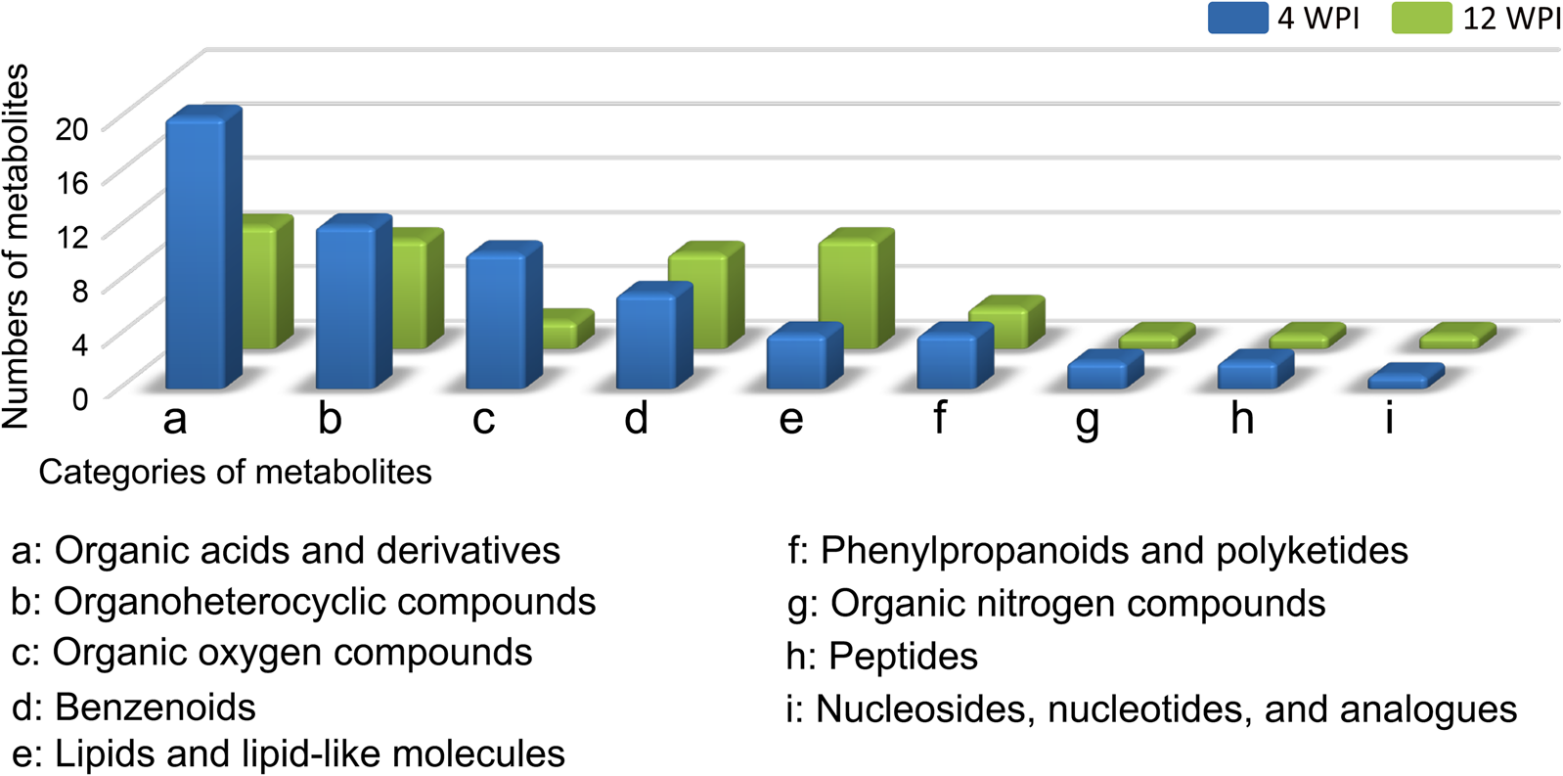
Categories and count of differential metabolites at 4 WPI and 12 WPI. The differential metabolites were categorized according to their structural features and the metabolite categories as per HMDB. Blue bars represent 4 WPI and green bars 12 WPI

The differential metabolites were categorized according to their structural features. The nomenclature referred to HMDB. In total, nine different subclasses of compounds were identified, including organic acids and derivatives, organoheterocyclic compounds, organic oxygen compounds, benzenoids, lipids and lipid-like molecules, phenylpropanoids and polyketides, organic nitrogen compounds, peptides, nucleosides, nucleotides and analogues, and other unclassified metabolites. By counting the different profiles of the numbers of these differential metabolites, between 4 and 12 WPI were revealed (Fig. 6). The organic acids and derivatives were predominant at 4 WPI, followed by organoheterocyclic compounds and organic oxygen compounds. At 12 WPI, the counts of these three kinds of compounds decreased, while lipids and lipid-like molecules increased.

The differential metabolites were hierarchically clustered according to their patterns of change and then visualized as heatmaps (Fig. 7 in ESI + mode and Additional file 7: Fig. S4 in ESI− mode). At 4 WPI, 61 differential metabolites were upregulated, while 29 were downregulated, in which almost all of the organic acids and derivatives and organic oxygen compounds were upregulated. At 12 WPI, 29 differential metabolites were upregulated, while 36 were downregulated, in which the organic acids and derivatives tended to be upregulated, while lipids and lipid-like molecules were downregulated.

**Fig. 7:**
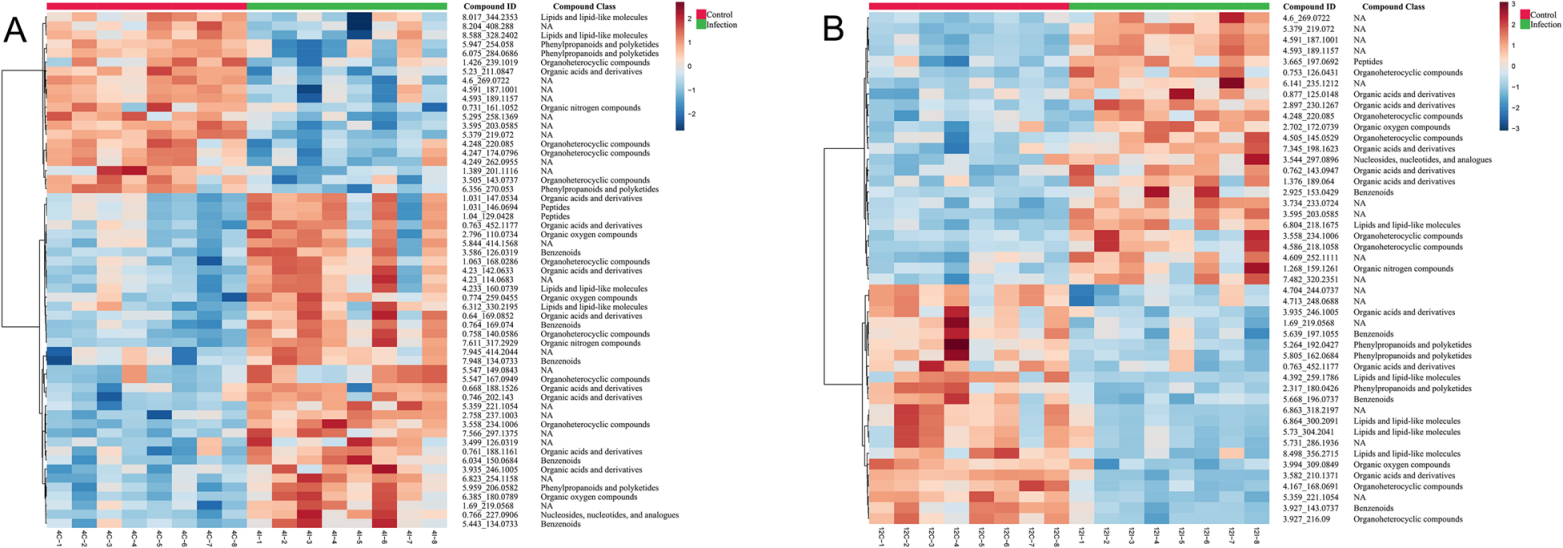
Heatmaps of differential metabolites at 4 WPI and 12 WPI in ESI + mode. The differential metabolites were hierarchically clustered according to their patterns of change and then visualized as heatmaps. **A** Results at 4 WPI in ESI + mode. **B** Results at 12 WPI in ESI + mode. Red bars on the top of each map indicate the control group and green bars the infection group. Metabolites are represented by rows. The colors reddish brown and blue indicate the increased and reduced metabolite intensity, respectively

These results revealed that the metabolic profile of the infection group, including the species, numbers and intensities of the differential metabolites, changed significantly at 4 and 12 WPI.

### Dysregulated metabolic pathways

**Fig. 8:**
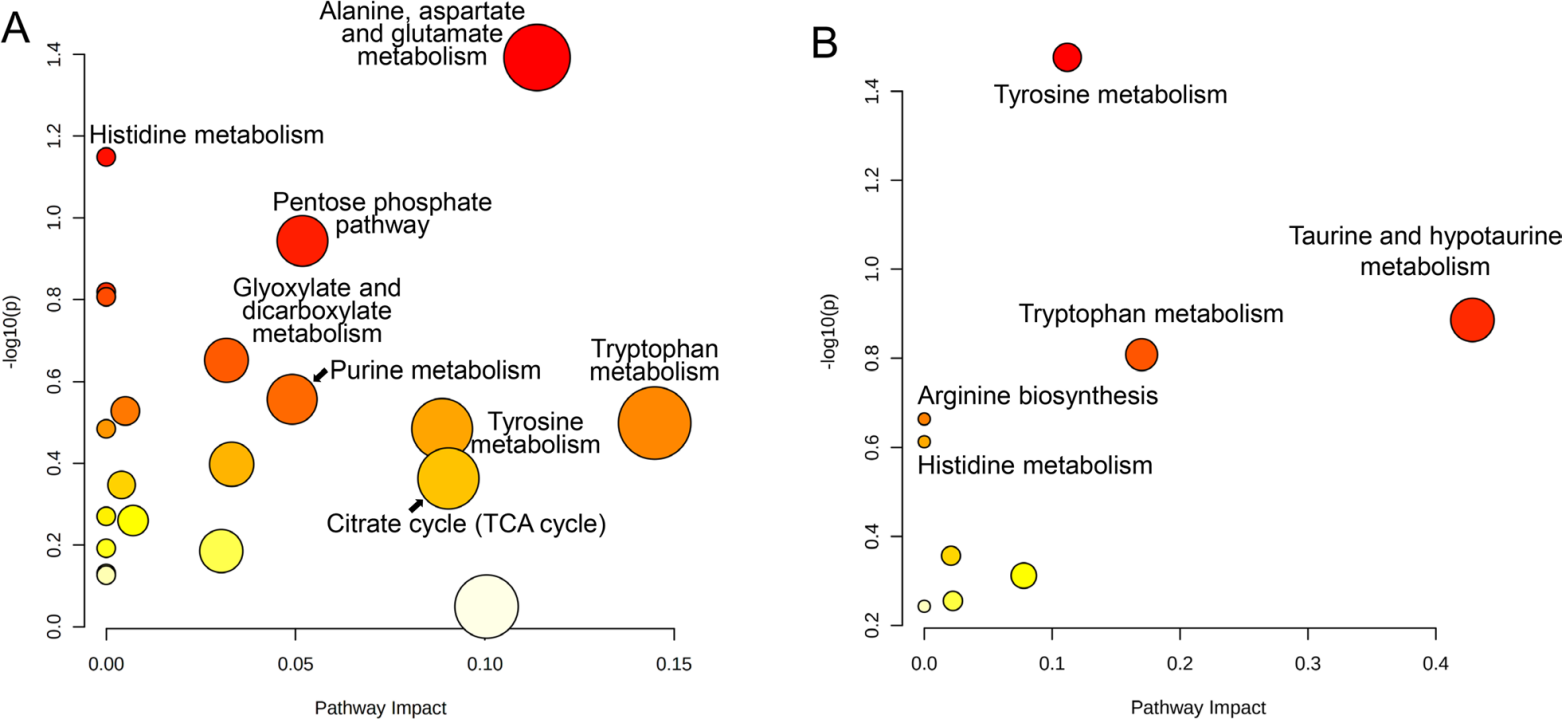
Dysregulated pathways at 4 WPI and 12 WPI. The pathway analysis was conducted using MetaboAnalyst 5.0. The deeper the color, the smaller the *p*-value. The larger the bubble size, the higher the pathway impact. **A** Dysregulated pathways at 4 WPI. **B** Dysregulated pathways at 12 WPI

The results of pathway analysis are shown in Fig. 8. At 4 WPI, the significantly affected metabolic pathways mainly contained alanine, aspartate and glutamate metabolism, the pentose phosphate pathway, glyoxylate and dicarboxylate metabolism, purine metabolism, histidine metabolism, tryptophan metabolism and tyrosine metabolism. At 12 WPI, the number of dysregulated metabolic pathways was less than that at 4 WPI. Tyrosine metabolism, taurine and hypotaurine metabolism and tryptophan metabolism were the markedly affected metabolic pathways. Information on differential metabolites involved in some dysregulated pathways is shown in Table 2. The detailed information on matched status of each dysregulated pathway is listed in Additional file 8.

**Table 2:** Information on some of the dysregulated pathways and related metabolites

Infection time	Pathway	Metabolite	Ionic strength change^a^		Mode
			4 WPI	12 WPI	
4 WPI	Alanine, aspartate and glutamate metabolism	Citrate	2.22 (0.041)	− 0.69 (*N*)	ESI−
		l-Glutamine	2.47 (0.048)	− 0.88 (*N*)	ESI+
		d-Glucosamine 6-phosphate	2.03 (0.017)	− 0.89 (*N*)	ESI+
	Pentose phosphate pathway	Δ-Gluconic acid δ-lactone	2.5 (0.009)	− 0.69 (*N*)	ESI−
		Gluconic acid	2.02 (0.003)	− 0.79 (*N*)	ESI−
	Tryptophan metabolism	5-Hydroxy-l-tryptophan	− 0.63 (0.008)	1.56 (*0.032*)	ESI+
		5-Hydroxyindole-3-acetic acid	− 0.57 (0.050)	1.34 (*N*)	ESI−
	Tyrosine metabolism	2,5-Dihydroxybenzaldehyde	1.94 (0.009)	− 0.5 (*N*)	ESI−
		Noradrenaline	1.67 (0.002)	− 0.85 (*N*)	ESI+
	Glyoxylate and dicarboxylate metabolism	Citrate	2.22 (0.041)	−0.69 (*N*)	ESI−
		l-Glutamine	2.47 (0.048)	− 0.88 (*N*)	ESI+
12 WPI	Tyrosine metabolism	Dl-Metanephrine	1.35 (*N*)	− 0.63 (0.030)	ESI+
		l-Dopa	− 0.97 (*N*)	1.67 (0.022)	ESI+
		2,5-Dihydroxybenzoate	− 0.91 (*N*)	1.63 (0.040)	ESI−
	﻿Tryptophan metabolism	5-Hydroxy-l-tryptophan	− 0.63 (*0.008*)	1.56 (0.032)	ESI+
		*N*-Acetylserotonin	− 0.8 (*N*)	5.48 (0.006)	ESI+
	Taurine and hypotaurine metabolism	Taurine	1.26 (*N*)	1.91 (0.036)	ESI+
			− 0.92 (*N*)	2.31 (0.015)	ESI−

ESI+ and ESI− = positive and negative mode in electrospray ionization

^a^Fold change, the ratio of intensity of *Infection* to *Control*; “−” denotes *downregulation*. Values in parentheses show *p*-values. Italic “*N*” means the FC is nonsignificant

### Screening and evaluation of potential biomarkers for VL

Through univariate and multivariate analyses, 12 metabolites varied at both 4 and 12 WPI and were selected as alternative biomarkers for subsequent evaluation. Through further univariate ROC tests, 7 of the 12 metabolites had an AUROC > 0.8 (Table 3), which showed relatively satisfactory performance in distinguishing between the infection group and control group. Hence, the seven metabolites were designated as a potential biomarker panel to be tested for VL diagnosis.

**Table 3:** Information on the screened potential biomarkers through PLS-DA and ROC tests

Mode	Compound ID	Compound name	AUROC		Ionic strength change^a^	
			4 WPI	12 WPI	4 WPI	12 WPI
ESI+	0.763_452.1177	3-Nitro-l-tyrosine	0.84	0.89	3.52	− 0.43
ESI+	3.558_234.1006	Dl-5-Methoxytryptophan	1	0.81	2.17	3.44
ESI+	3.595_203.0585	1-(2,4-Dihydroxyquinolin-3-yl)ethan-1-one	1	1	− 0.31	3.25
ESI+	4.248_220.085	5-Hydroxy-l-tryptophan	0.91	0.86	− 0.63	1.56
ESI+	4.591_187.1001	2-[(Dimethylamino)methylidene]indan-1-one	0.94	1	− 0.35	6.82
ESI+	4.593_189.1157	*N*-Benzyl-4-piperidone	0.97	1	− 0.26	9.87
ESI−	5.562_269.0724	*N*-(1,1-Dioxothiolan-3-yl)-4-methoxybenzamide	0.97	1	− 0.32	6.84

*ESI+ and ESI−* = positive and negative mode in electrospray ionization

^a^Fold change, the ratio of intensity of *Infection* to *Control*, “−” denotes *downregulation*

**Fig. 9:**
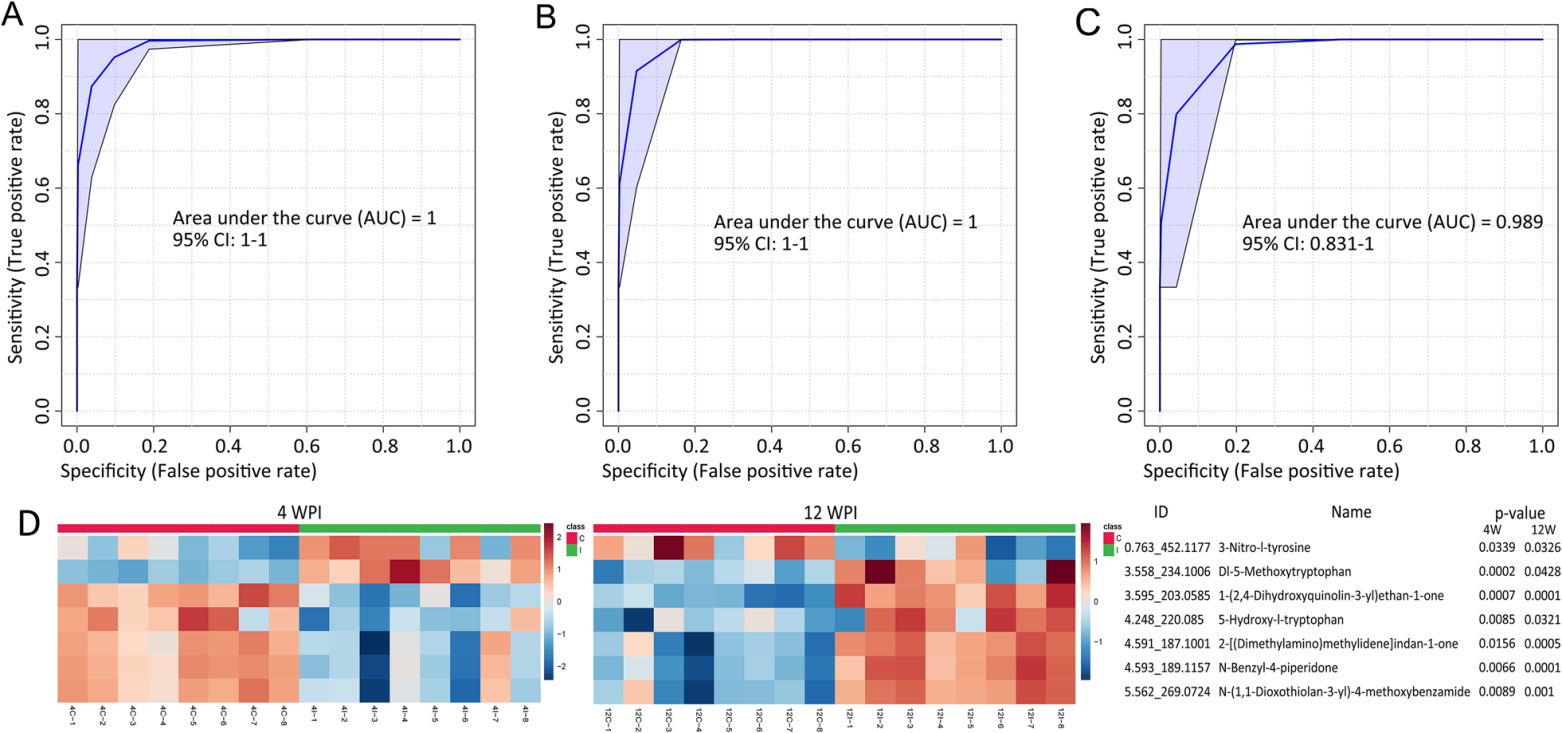
Heatmaps and ROC curve for assessing the diagnostic potential of the selected biomarker panel. The closer the AUC was to 1.0, the better the effectiveness of the predictive diagnosis. **A** ROC curve of the biomarker panel at 4 WPI. **B** ROC curve of biomarker panel at 12 WPI. **C** ROC curve of the biomarker panel for 4 WPI vs. 12 WPI. **D** Ionic strength changes of the seven potential biomarkers at 4 WPI and 12 WPI were visualized as heatmaps. Each row in the heatmaps at 4 WPI and 12 WPI corresponded to the same compound ID and name on the right

The specificity and sensitivity of the biomarker panel in detecting VL were then assessed by ROC curve analysis. For identifying the infection group at 4 or 12 WPI, the sensitivity and specificity were very high, as the AUROCs were almost 1 (Fig. 9A, B). In addition, the performance of the biomarker panel was also satisfactory in distinguishing infection groups between 4 and 12 WPI, as the AUROC was 0.989 with a 95% confidence interval from 0.831 to 1 (Fig. 9C). These results suggested that the selected biomarker panel has the potential for VL diagnosis and course monitoring. The heatmaps in Fig. 9D showed the ionic strength changes of the seven potential biomarkers at 4 WPI and 12 WPI.

## Discussion

According to the categorized results of differential metabolites and heatmaps (Figs. 6, 7), we found that the organic acids and derivatives accounted for a significant portion of the differential metabolites, especially almost all of the organic acids and derivatives and organic oxygen compounds that were upregulated at 4 WPI. Meanwhile, the enrichment analyses of metabolic pathways showed that some amino acid metabolic pathways were affected most significantly at 4 WPI and 12 WPI, including alanine, aspartate and glutamate metabolism, taurine and hypotaurine metabolism, tryptophan metabolism and tyrosine metabolism. This reflected that there was remarkable abnormality in the amino acid metabolism of the infected hamsters. According to our previous research on *Leishmania* hamster models [25], 4 WPI was still a relatively earlier stage among the whole infectious period, at which point the parasites proliferated rapidly. *Leishmania* has a huge demand for energy during the earlier growth period, and amino acids are an important carbon source for *Leishmania*. Although *Leishmania* can synthesize some amino acids de novo, many of them are auxotrophic [36] and must be obtained from the host’s environment. This might be the cause of the disorder of amino acid metabolism in the host. Amino acid metabolism occurs primarily in the liver, and in this study, parasite enrichment in the liver existed at 4 WPI according to the results of pathological sections and real-time PCR. Combined with our recent study on serum metabolomics [25], we found that *Leishmania* enrichment in the liver at an early stage can cause damage to the liver and distinctly disturb the energy metabolism of the host, particularly lipid and amino acid metabolism.

The numbers of identified differential metabolites and pathway analysis showed that the disturbance of metabolism at 12 WPI was obviously decreased compared with that at 4 WPI (Table 1; Fig. 8). In our VL golden hamster model, compared with 4 WPI, 12 WPI is the mid- to late period, at which time more amastigotes and granulomas could be observed in visceral organs (Figs. 1, 2). There is evidence that the amastigote growth in the granuloma microenvironment is highly constrained [37]. The increase of parasite burden at late stage generally levels off according to the research of other VL mouse model [38, 39]. Many semi-quiescent amastigotes are in a distinct stringent metabolic state with a global decrease in energy consuming processes [14, 37, 40]. Therefore, we speculated that the reduced metabolic disturbance of urine at 12 WPI may be linked to a slow growth state of *Leishmania* in a lesion environment at the mid- to late period.

In this study, alanine, aspartate and glutamate metabolism were the significantly affected metabolic pathways at 4 WPI. It is well established that together with alanine and aspartate, glutamate is directly involved in providing the Krebs cycle with intermediates [41]. It also participated in the differentiation process from epimastigotes to metacyclic trypomastigotes of *Trypanosoma cruzi* [42]. Among the metabolites involved in this pathway, l-glutamine and d-glucosamine 6-phosphate were upregulated at 4 WPI according to the ion strength calculation (Table 2). It has been demonstrated that *Leishmania* amastigotes are highly dependent on mitochondrial metabolism for the de novo synthesis of glutamate and glutamine [14]. In addition, research has shown that glutamine can potentiate parasite clearance through the development of a more effective anti-*Leishmania* adaptive immune response, and it also demonstrated an upregulation of the genes associated with glutaminolysis as well as an increase in glutamine consumption of *Leishmania donovani*-infected macrophages [43]. Thus, upregulation of the glutamine content in urine is likely related to the common nutrient demand of *Leishmania* survival and immune cells as well as the potentiation of the immune response of host at the earlier infective stage. d-glucosamine 6-phosphate is the intermediate metabolite from glutamine to biosynthesis of hexosamines. It has been reported that the biosynthesis of hexosamines, such as *N*-acetylglucosamine, was essential to *Leishmania* growth and virulence [44], while no hexosamines were detected in urine samples in this study; perhaps the metabolic analysis from other biospecimens are needed. At 4 WPI, PPP is also an affected pathway. PPP is a key pathway in trypanosomatids, which primally generates nicotinamide adenine dinucleotide phosphate (NADPH) as a source of reducing power and other products that feed diverse parts of the metabolism [45]. The differential metabolites involved in this pathway including d-gluconic acid, d-glucono-1,5-lactone and d-ribulose 5-phosphate (d-ribulose 5-phosphate was identified as level 4 for its lower confidence level and is not shown in Table 2), and they were all upregulated (Table 2). They are intermediate metabolites of the oxidative branch in PPP, and it has been reported that PPP enables glucose to serve as a source of ribose-5-phosphate in nucleotide biosynthesis[46]. Therefore, the upregulated oxidative branch of the PPP probably supplies parasites with ribose-5-phosphate (R5P), which produces RNA and DNA, and NADPH for use as a cellular reductant in cellular metabolism. Recently, PPP has become a very attractive and viable metabolic pathway in drug target research of leishmaniasis, especially some enzymes that are involved in it [47–50], which is expected to lead to the development of novel drugs against VL.

Then, we found that tryptophan metabolism was affected observably at 4 WPI and 12 WPI. Tryptophan is an essential amino acid in mammals and plays an important role in immunomodulation of the host [51, 52]. Intracellular L-tryptophan is catabolized through two major pathways: (1) conversion into kynurenine via indoleamine 2,3-dioxygenase or tryptophan 2,3-dioxygenase [53] and (2) conversion into an intermediary metabolite 5-hydroxytryptophan (5-HTP) via tryptophan hydroxylase (TPH). In this study, the differential metabolites detected at 4 WPI or 12 WPI were involved in the 5-HTP pathway. Significant differences in metabolites that matched the kynurenine pathway were not detected. In addition, the differential metabolites matching the tryptophan pathway at 4 WPI were all down- and upregulated at 12 WPI. Generally, the decrease in metabolites reflected reduced biosynthesis or enhanced catabolism. *Leishmania* is auxotrophic for tryptophan and lacks the genes for its biosynthesis [54, 55]. *Leishmania donovani* was reported to consume a large amount of tryptophan during infection in vitro [12], and catabolism in the tryptophan pathway produces NAD+, which can help to maintain redox homeostasis. In addition, tryptophan depletion in host cells suppressed T-cell proliferation and thereby promoted the chronicity of disease [51, 56]. Whether the downregulation of the metabolites in 5-HTP pathway was related to tryptophan consumption in *Leishmania* infection and thereby attenuated the host’s immunity at the earlier infectious stage needs further experimental validation, especially studies on the role of the 5-HTP pathway in VL progression.

Moreover, in tyrosine metabolism, we found that 3-nitrotyrosine (3-nitro-l-tyrosine) varied significantly at both 4 and 12 WPI. From Table 3, compared with the control groups, it was significantly upregulated more than three times at 4 WPI, while it was downregulated more than two times at 12 WPI. 3-Nitrotyrosine, also known as nitrotyrosine, is a product of tyrosine nitration mediated by reactive nitrogen species such as peroxynitrite anion and nitrogen dioxide. 3-Nitrotyrosine is a well-established stable oxidative stress biomarker and a good predictor of the progression of some diseases, such as cardiovascular and neurodegenerative diseases [57–59]. The enhanced production of 3-nitrotyrosine was closely related to the enhanced microbicidal activity in *Leishmania*-infected macrophages [60]. A previous study revealed that 3-nitrotyrosine formation is a relevant indicator of antiparasitic activity [61]. Hence, the upregulated 3-nitrotyrosine at 4 WPI in urine may be related to the increased antiparasitic activity of infected macrophages at the earlier infection period, and the downregulation at 12 WPI was probably related to the progression to the late chronic infection stage of VL. Certainly, these inferences still need in-depth validation. In addition, the metabolite taurine involved in taurine and hypotaurine metabolism was upregulated at 12 WPI. Taurine, one of the most abundant amino acids, possesses anti-inflammatory and immunoregulatory properties [62] and protects against various types of hepatic damage [63, 64]. However, the role of taurine in the VL process and whether its upregulation is related to the self-limitation of pathological lesions in liver in this study are unknown.

In view of availability and compliance, urine is the type of specimen that can be sampled noninvasively in high quantities. The abundance of terminal metabolites of the body’s biochemical metabolism also makes urine a valuable sample in biomarker searching [23, 27], and biomarker discovery allows for a better understanding of the underlying pathogenic mechanism. In this study, 12 metabolites were identified that varied significantly at both 4 and 12 WPI. Through univariate ROC analysis, 7 of the 12 metabolites showed relatively satisfactory performance in distinguishing between the infection group and the control group, with an AUC > 0.8 (Table 3). Hence, the seven metabolites were designated as a potential biomarker panel to be tested for diagnosis. The results of the multivariable ROC test suggested that the selected biomarker panel had a strong ability to distinguish the infection groups from the control groups at 4 and 12 WPI (Fig. 9A, B). In addition, the capacity of the biomarker panel to separate the infection groups between 4 and 12 WPI was also demonstrated (Fig. 9C). These analyses showed that the biomarker panel of seven urine metabolites may have potential in VL diagnosis and infectious stage monitoring, which would be worth further validation.

According to the ionic strength change (Table 3; Fig. 9D), the five of seven metabolites were downregulated at 4 WPI and upregulated drastically at 12 WPI, whose same changing pattern implied that these metabolites may be involved in close metabolic pathways. However, most of these metabolites lacked detailed information in the existing compound library. Among the other two metabolites, Dl-5-methoxytryptophan (5-MPT) was upregulated at both 4 WPI and 12 WPI. 5-MPT is an endogenous metabolite that is produced in fibroblasts by a novel tryptophan metabolic pathway [65] and has been confirmed to be capable of defending against excessive systemic inflammatory responses and sepsis [66]. It could also contribute to reducing tumour growth and metastasis as well as reducing intimal hyperplasia in vascular disease [65, 67, 68]. However, to date, we have not seen any research about 5-MPT in leishmaniasis. In recent studies, in addition to being diagnostic and therapeutic biomarkers of disease, 5-MPT may also be a valuable lead compound, and its synthetase TPH-1 may serve as a target marker of some diseases [68–71]. Hence, further targeted validation of 5-MPT is necessary for its diagnostic potential. Meanwhile, research on the biological function of 5-MPT and the expression of its synthetase would be useful to understand the role of 5-MPT in VL progression.

## Conclusions

In this study, urine metabonomics analysis revealed that the metabolism of golden hamsters was disturbed significantly after *Leishmania* infection. Thus, amino acid metabolism was greatly affected at both infection time points. It also presented distinct metabolic characteristics at the early and late stages of VL progression. The number of disturbed metabolites and metabolic pathways at 4 WPI was obviously greater than that at 12 WPI. In addition to amino acid metabolism, the influenced metabolism also involved carbohydrate and nucleotide metabolism at 4 WPI. This may be related to the differences in the nutrient demands of parasites and/or host immune responses at earlier and mid- to late stages of VL. In addition, the results of this study were quite different from those of our previous metabonomics study on serum, which reflected that lipid metabolism was the most influenced pathway after infection. Together, metabonomics studies from different biological samples help with the overall understanding of the metabolic characteristics of VL. The seven metabolites were screened as a potential biomarker panel that has good potential in VL diagnosis in this study and is worthy of further validation, which is expected to contribute to the development of a novel diagnostic approach and course monitoring of VL. Our study further offers more abundant metabolic information on *Leishmania*-infected hosts, which we hope provides a novel direction for future research on the molecular mechanisms of *Leishmania* infection.

### Supplementary Information

**Additional file 1: Table S1.** Real-time PCR condition for *Leishmania* load.

**Additional file 2:** Detailed method for urine sample pretreatment and the component of inner standards.

**Additional file 3: Figure S1.** Real-time PCR standard curve.

**Additional file 4: Figure S2.** TIC curves and PCA plots of QC samples. (A) TIC curves of QC samples were highly overlapped. The left represents the ESI + mode, and the right represents the ESI− mode. (B) PCA plots of QC samples. Black arrows point to the points of QC samples that were tightly aggregated.

**Additional file 5: Figure S3.** PLS-DA plots of experimental samples in ESI− mode. (A) Control vs. infection at 4 WPI. R2 = 1.00, Q2 = 0.78. (B) Control vs. infection at 12 WPI. R2 = 0.99, Q2 = 0.62. (C) 4 WPI vs. 12 WPI of the infection group. R2 = 0.99, Q2 = 0.68.

**Additional file 6:** Detailed information of the total differential metabolites in this study.

**Additional file 7:** Heatmaps of differential metabolites at 4 WPI and 12 WPI in ESI− mode. (A) Results at 4 WPI in ESI− mode. (B) Results at 12 WPI in ESI− mode.

**Additional file 8:** Detailed information about matched status of each dysregulated pathway.

## Data Availability

All data generated or analyzed during this study are included in this published article and its supplementary information files. The raw data had been uploaded to Metabolights, the accession number is MTBLS8191. Please find it at https://www.ebi.ac.uk/metabolights/MTBLS8191.

## Abbreviations

WPI: Week post infection
VL: Visceral leishmaniasis
PLS-DA: Partial least squares-discriminant analysis
ROC: Receiver-operating characteristic
PCA: Principal component analysis
FC: Fold change
VIP: Variable importance for the projection
ESI+ and ESI−: Positive and negative mode in electrospray ionization

## References

[1] WHO. Global leishmaniasis surveillance, 2017–2018, and first report on 5 additional indicators. WHO Weekly Epidemiological Record, No 25, 2020. Available from: https://www.who.int/publications/i/item/who-wer9525.

[2] Burza S, Croft SL, Boelaert M (2018). Leishmaniasis. Lancet.

[3] Alvar J, Vélez ID, Bern C, Herrero M, Desjeux P, Cano J (2012). Leishmaniasis worldwide and global estimates of its incidence. PLoS ONE.

[4] Zhou Z, Li Y, Zhang Y, Li S (2020). Prevalence of visceral leishmaniasis in China during 2015–2018. Chin J Parasitol Parasit Dis.

[5] Yuan D, Qin H, Zhang J, Liao L, Chen Q, Chen D (2017). Phylogenetic analysis of HSP70 and cyt *b* gene sequences for Chinese isolates and ultrastructural characteristics of Chinese *Leishmania* sp. Parasitol Res.

[6] Yuan D, Qin H, Chen D, Chen J (2021). Genetic diversity analysis of Chinese *Leishmania* isolates and development of *L. **donovani* complex-specific markers by RAPD. BMC Infect Dis.

[7] Sundar S, Rai M (2002). Laboratory diagnosis of visceral leishmaniasis. Clin Diagn Lab Immunol.

[8] Sundar S, Singh OP (2018). Molecular diagnosis of visceral Leishmaniasis. Mol Diagn Ther.

[9] Ponte-Sucre A, Gamarro F, Dujardin JC, Barrett MP, López-Vélez R, García-Hernández R (2017). Drug resistance and treatment failure in leishmaniasis: A 21st century challenge. PLoS Negl Trop Dis.

[10] Podinovskaia M, Descoteaux A (2015). *Leishmania* and the macrophage: a multifaceted interaction. Future Microbiol.

[11] Séguin O, Descoteaux A (2016). *Leishmania*, the phagosome, and host responses: the journey of a parasite. Cell Immunol.

[12] Westrop GD, Williams RA, Wang L, Zhang T, Watson DG, Silva AM (2015). Metabolomic analyses of *Leishmania* reveal multiple species differences and large differences in amino acid metabolism. PLoS ONE.

[13] Castilho-Martins EA, Canuto GAB, Muxel SM, daSilva MFL, Floeter-Winter LM, Del Aguila C (2015). Capillary electrophoresis reveals polyamine metabolism modulation in *Leishmania* (*Leishmania*) *amazonensis* wild-type and arginase-knockout mutants under arginine starvation. Electrophoresis.

[14] Saunders EC, Ng WW, Kloehn J, Chambers JM, Ng M, McConville MJ (2014). Induction of a stringent metabolic response in intracellular stages of *Leishmania **mexicana* leads to increased dependence on mitochondrial metabolism. PLoS Pathog.

[15] Muxel SM, Mamani-Huanca M, Aoki JI, Zampieri RA, Floeter-Winter LM, López-Gonzálvez Á (2019). Metabolomic profile of BALB/c macrophages infected with *Leishmania **amazonensis*: deciphering l-arginine metabolism. Int J Mol Sci.

[16] Mamani-Huanca M, Muxel SM, Acuña SM, Floeter-Winter LM, Barbas C, López-Gonzálvez Á. Metabolomic reprogramming of C57BL/6-macrophages during early infection with *L. amazonensis.* Int J Mol Sci. 2021;22(13). 10.3390/ijms22136883 PMC8267886 34206906

[17] Lamour SD, Choi BS, Keun HC, Müller I, Saric J (2012). Metabolic characterization of *Leishmania major* infection in activated and nonactivated macrophages. J Proteome Res.

[18] Berg M, Vanaerschot M, Jankevics A, Cuypers B, Maes I, Mukherjee S (2013). Metabolic adaptations of *Leishmania **donovani* in relation to differentiation, drug resistance, and drug pressure. Mol Microbiol.

[19] Vincent IM, Weidt S, Rivas L, Burgess K, Smith TK, Ouellette M (2014). Untargeted metabolomic analysis of miltefosine action in *Leishmania infantum* reveals changes to the internal lipid metabolism. Int J Parasitol Drugs Drug Resist.

[20] Canuto GA, Castilho-Martins EA, Tavares MF, Rivas L, Barbas C, López-Gonzálvez Á (2014). Multi-analytical platform metabolomic approach to study miltefosine mechanism of action and resistance in *Leishmania*. Anal Bioanal Chem.

[21] Canuto GA, Castilho-Martins EA, Tavares M, López-Gonzálvez A, Rivas L, Barbas C (2012). CE-ESI-MS metabolic fingerprinting of *Leishmania* resistance to antimony treatment. Electrophoresis.

[22] Rojo D, Canuto GA, Castilho-Martins EA, Tavares MF, Barbas C, López-Gonzálvez Á (2015). A multiplatform metabolomic approach to the basis of antimonial action and resistance in *Leishmania infantum*. PLoS ONE.

[23] Bujak R, Struck-Lewicka W, Markuszewski MJ, Kaliszan R (2015). Metabolomics for laboratory diagnostics. J Pharm Biomed Anal.

[24] Vargas DA, Prieto MD, Martínez-Valencia AJ, Cossio A, Burgess KEV, Burchmore RJS (2019). Pharmacometabolomics of meglumine antimoniate in patients with cutaneous Leishmaniasis. Front Pharmacol.

[25] Qin H, Zhang J, Dong K, Chen D, Yuan D, Chen J (2022). Metabolic characterization and biomarkers screening for visceral leishmaniasis in golden hamsters. Acta Trop.

[26] Kloehn J, Blume M, Cobbold SA, Saunders EC, Dagley MJ, McConville MJ (2016). Using metabolomics to dissect host–parasite interactions. Curr Opin Microbiol.

[27] Khamis MM, Adamko DJ, El-Aneed A (2017). Mass spectrometric based approaches in urine metabolomics and biomarker discovery. Mass Spectrom Rev.

[28] Das S, Saha T, Shaha C (2021). Tissue/biofluid specific molecular cartography of *Leishmania **donovani* infected BALB/c mice: deciphering systemic reprogramming. Front Cell Infect Microbiol..

[29] Zhang A, Sun H, Wu X, Wang X (2012). Urine metabolomics. Clin Chim Acta.

[30] Loría-Cervera EN, Andrade-Narváez FJ (2014). Animal models for the study of leishmaniasis immunology. Rev Inst Med Trop Sao Paulo.

[31] Wang X, Lv H, Zhang G, Sun W, Zhou D, Jiao G (2008). Development and validation of a ultra performance LC-ESI/MS method for analysis of metabolic phenotypes of healthy men in day and night urine samples. J Sep Sci.

[32] Rolão N, Cortes S, Rodrigues OR, Campino L (2004). Quantification of *Leishmania infantum* parasites in tissue biopsies by real-time polymerase chain reaction and polymerase chain reaction-enzyme-linked immunosorbent assay. J Parasitol.

[33] Wen B, Mei Z, Zeng C, Liu S (2017). metaX: a flexible and comprehensive software for processing metabolomics data. BMC Bioinformatics.

[34] Chong J, Wishart DS, Xia J (2019). Using MetaboAnalyst 4.0 for comprehensive and integrative metabolomics data analysis. Curr Protoc Bioinformatics..

[35] Schymanski EL, Jeon J, Gulde R, Fenner K, Ruff M, Singer HP (2014). Identifying small molecules via high resolution mass spectrometry: communicating confidence. Environ Sci Technol.

[36] McConville MJ, de Souza D, Saunders E, Likic VA, Naderer T (2007). Living in a phagolysosome; metabolism of *Leishmania* amastigotes. Trends Parasitol.

[37] Kloehn J, Saunders EC, O'Callaghan S, Dagley MJ, McConville MJ (2015). Characterization of metabolically quiescent *Leishmania* parasites in murine lesions using heavy water labeling. PLoS Pathog.

[38] Melby PC, Tabares A, Restrepo BI, Cardona AE, McGuff HS, Teale JM (2001). *Leishmania **donovani*: evolution and architecture of the splenic cellular immune response related to control of infection. Exp Parasitol.

[39] Engwerda CR, Ato M, Kaye PM (2004). Macrophages, pathology and parasite persistence in experimental visceral leishmaniasis. Trends Parasitol.

[40] Saunders EC, Sernee MF, Ralton JE, McConville MJ (2021). Metabolic stringent response in intracellular stages of *Leishmania*. Curr Opin Microbiol.

[41] Paes LS, Galvez Rojas RL, Daliry A, Floeter-Winter LM, Ramirez MI, Silber AM (2008). Active transport of glutamate in *Leishmania* (*Leishmania*) *amazonensis*. J Eukaryot Microbiol.

[42] Contreras VT, Salles JM, Thomas N, Morel CM, Goldenberg S (1985). In vitro differentiation of *Trypanosoma **cruzi* under chemically defined conditions. Mol Biochem Parasitol.

[43] Ferreira C, Mesquita I, Barbosa AM, Osório NS, Torrado E, Beauparlant CJ (2020). Glutamine supplementation improves the efficacy of miltefosine treatment for visceral leishmaniasis. PLoS Negl Trop Dis.

[44] Naderer T, Wee E, McConville MJ (2008). Role of hexosamine biosynthesis in *Leishmania* growth and virulence. Mol Microbiol.

[45] Kovářová J, Barrett MP (2016). The pentose phosphate pathway in parasitic trypanosomatids. Trends Parasitol.

[46] Maugeri DA, Cazzulo JJ, Burchmore RJ, Barrett MP, Ogbunude PO (2003). Pentose phosphate metabolism in *Leishmania **mexicana*. Mol Biochem Parasitol.

[47] Dhumal TT, Kumar R, Paul A, Roy PK, Garg P, Singh S (2022). Molecular explorations of the *Leishmania **donovani* 6-phosphogluconolactonase enzyme, a key player in the pentose phosphate pathway. Biochimie.

[48] Narsimulu B, Qureshi R, Jakkula P, Are S, Qureshi IA (2022). Biophysical and structural characterization of ribulose-5-phosphate epimerase from *Leishmania **donovani*. ACS Omega.

[49] Pinho N, Bombaça AC, Wiśniewski JR, Dias-Lopes G, Saboia-Vahia L, Cupolillo E (2022). Nitric oxide resistance in *Leishmania* (*Viannia*) *braziliensis* involves regulation of glucose consumption, glutathione metabolism and abundance of pentose phosphate pathway enzymes. Antioxidants (Basel)..

[50] Jakkula P, Narsimulu B, Qureshi IA (2021). Biochemical and structural insights into 6-phosphogluconate dehydrogenase from *Leishmania **donovani*. Appl Microbiol Biotechnol.

[51] Makala LH (2012). The role of indoleamine 2, 3 dioxygenase in regulating host immunity to *Leishmania* infection. J Biomed Sci.

[52] Grohmann U, Bronte V (2010). Control of immune response by amino acid metabolism. Immunol Rev.

[53] Moffett JR, Namboodiri MA (2003). Tryptophan and the immune response. Immunol Cell Biol.

[54] Burchmore RJ, Barrett MP (2001). Life in vacuoles–nutrient acquisition by Leishmania amastigotes. Int J Parasitol.

[55] Opperdoes FR, Coombs GH (2007). Metabolism of *Leishmania*: proven and predicted. Trends Parasitol.

[56] Gangneux JP, Poinsignon Y, Donaghy L, Amiot L, Tarte K, Mary C (2013). Indoleamine 2,3-dioxygenase activity as a potential biomarker of immune suppression during visceral leishmaniasis. Innate Immun.

[57] Darwish RS, Amiridze N, Aarabi B (2007). Nitrotyrosine as an oxidative stress marker: evidence for involvement in neurologic outcome in human traumatic brain injury. J Trauma.

[58] Campolo N, Issoglio FM, Estrin DA, Bartesaghi S, Radi R (2020). 3-Nitrotyrosine and related derivatives in proteins: precursors, radical intermediates and impact in function. Essays Biochem.

[59] Thomson L, Tenopoulou M, Lightfoot R, Tsika E, Parastatidis I, Martinez M (2012). Immunoglobulins against tyrosine-nitrated epitopes in coronary artery disease. Circulation.

[60] Kawakami NY, Tomiotto-Pellissier F, Cataneo AH, Orsini TM, Thomazelli AP, Panis C (2016). Sodium nitroprusside has leishmanicidal activity independent of iNOS. Rev Soc Bras Med Trop.

[61] Linares E, Giorgio S, Mortara RA, Santos CX, Yamada AT, Augusto O (2001). Role of peroxynitrite in macrophage microbicidal mechanisms in vivo revealed by protein nitration and hydroxylation. Free Radic Biol Med.

[62] De Luca A, Pierno S, Camerino DC (2015). Taurine: the appeal of a safe amino acid for skeletal muscle disorders. J Transl Med.

[63] Gentile CL, Nivala AM, Gonzales JC, Pfaffenbach KT, Wang D, Wei Y (2011). Experimental evidence for therapeutic potential of taurine in the treatment of nonalcoholic fatty liver disease. Am J Physiol Regul Integr Comp Physiol.

[64] Yu YR, Ni XQ, Huang J, Zhu YH, Qi YF (2016). Taurine drinking ameliorates hepatic granuloma and fibrosis in mice infected with *Schistosoma japonicum*. Int J Parasitol Drugs Drug Resist.

[65] Cheng HH, Kuo CC, Yan JL, Chen HL, Lin WC, Wang KH (2012). Control of cyclooxygenase-2 expression and tumorigenesis by endogenous 5-methoxytryptophan. Proc Natl Acad Sci USA.

[66] Wang YF, Hsu YJ, Wu HF, Lee GL, Yang YS, Wu JY (2016). Endothelium-derived 5-methoxytryptophan is a circulating anti-inflammatory molecule that blocks systemic inflammation. Circ Res.

[67] Wu KK, Cheng HH, Chang TC (2014). 5-methoxyindole metabolites of l-tryptophan: control of COX-2 expression, inflammation and tumorigenesis. J Biomed Sci.

[68] Ho YC, Wu ML, Su CH, Chen CH, Ho HH, Lee GL (2016). A novel protective function of 5-methoxytryptophan in vascular injury. Sci Rep.

[69] Chen DQ, Cao G, Chen H, Argyopoulos CP, Yu H, Su W (2019). Identification of serum metabolites associating with chronic kidney disease progression and anti-fibrotic effect of 5-methoxytryptophan. Nat Commun.

[70] Wu KK, Kuo CC, Yet SF, Lee CM, Liou JY (2020). 5-methoxytryptophan: an arsenal against vascular injury and inflammation. J Biomed Sci.

[71] Wu KK (2021). Cytoguardin: a tryptophan metabolite against cancer growth and metastasis. Int J Mol Sci.

