# Supplementary material for: Metabolomics analysis of visceral leishmaniasis based on urine of golden hamsters
Source: Parasit Vectors. 2023 Aug 30;16:304. doi: 10.1186/s13071-023-05881-3 (PMC10469881; doi:10.1186/s13071-023-05881-3)
Supplement: Supplementary file 1 — Additional file 1: Table S1. Real-time PCR condition for Leishmania load. [file 13071_2023_5881_MOESM1_ESM.docx]

**Table S1.** Real-time PCR condition for *Leishmania* load.

(A) Reaction system.

| Component | Loading |
| --- | --- |
| 2×SuperReal PreMix (Probe) | 10μL |
| Forward primer | 0.6μL |
| Reverse Primer | 0.6μL |
| Fluorescent probe | 0.4μL |
| DNA template | 1μL |
| RNase-free ddH_2_O | 7.4μL |

(B) PCR Program

| Processes | Temperature | Time | Cycle | Signal acquisition |
| --- | --- | --- | --- | --- |
| Predegeneration | 95℃ | 15min | 1 | No |
| Amplification | 95℃ | 3s | 40 | No |
|  | 60℃ | 30s |  | Yes |

Forward primer (5’ to 3’): GGTTAGCCGATGGTGGTCTT

Reverse primer (5’ to 3’): GCTATATCATATGTCCAAGCACTTACCT

Fluorescent probe (5’ to 3’): FAM-ACCACCTAAGGTCAACCC-BHQ1
