# Supplementary material for: Metabolomics analysis of visceral leishmaniasis based on urine of golden hamsters
Source: Parasit Vectors. 2023 Aug 30;16:304. doi: 10.1186/s13071-023-05881-3 (PMC10469881; doi:10.1186/s13071-023-05881-3)
Supplement: Supplementary file 2 — Additional file 2: Detailed method for urine sample pretreatment and the component of inner standards. [file 13071_2023_5881_MOESM2_ESM.docx]

**1. Method for sample pretreatment**

Urine samples were slowing unfreezed at 4℃. After that, 100 μL of each sample were took into a 96-well plate. Then 300 μL precooled extracting solution (containg methyl alcohol and acetonitrile at the volume ratio of 2:1) were added. Next, the internal standard 1 and internal standard 2 were added**. The sample wells were vortexed for 1 min, standing at -20℃ for 2 hours, then at 4℃ centrifuged at 4000 rpm for 30 min. Take the supernatant into vials for LC-MS processing.

Ten microliter of mixture were took out from each sample well to blend and form a QC sample, for assessment of the repeatability and stability of LC-MS processing.

****The component of inner standards.**

Inner standard 1:

D3-L-Methionine (100 ppm, TRC, Canada), 13C9-Phenylalanine (100ppm, CIL, USA), D6-L-2-Aminobutyric Acid(100ppm, TRC, Canada), D4-L-Alanine (100ppm, TRC, Canada), 13C4-L-Threonine (100ppm, CIL, USA), D3-L-Aspartic Acid (100ppm, TRC, Canada), 13C6-L-Arginine (100ppm, CIL, USA).

Inner standard 2:

SPLASHTM Lipidomix Mass Spec Standard 330707 (Avanti Polar Lipids, USA). The storage concentration of each lipid standard is as follows:

LPC 18:1(d7), 25 μg/mL; LPE 18:1(d7), 5 μg/mL; PC 15:0–18:1(d7), 160 μg/mL; PE 15:0–18:1(d7), 5 μg/mL; PG 15:0–18:1(d7), 30 μg/mL; PS 15:0–18:1(d7), 5 μg/mL; PI 15:0–18:1(d7), 10 μg/mL; PA 15:0–18:1(d7), 7 μg/mL; SM d18:1–18:1(d9), 30 μg/mL; cholesterol(d7), 100 μg/mL; CE 18:1(d7), 350 μg/mL; MG 18:1(d7), 2 μg/mL; DG 15:0–18:1(d7), 10μg/mL; and TG 15:0–18:1(d7)–15:0, 55 μg/mL
