# Supplementary figures and images for: Metabolomics analysis of visceral leishmaniasis based on urine of golden hamsters
Source: Parasit Vectors. 2023 Aug 30;16:304. doi: 10.1186/s13071-023-05881-3 (PMC10469881; doi:10.1186/s13071-023-05881-3)

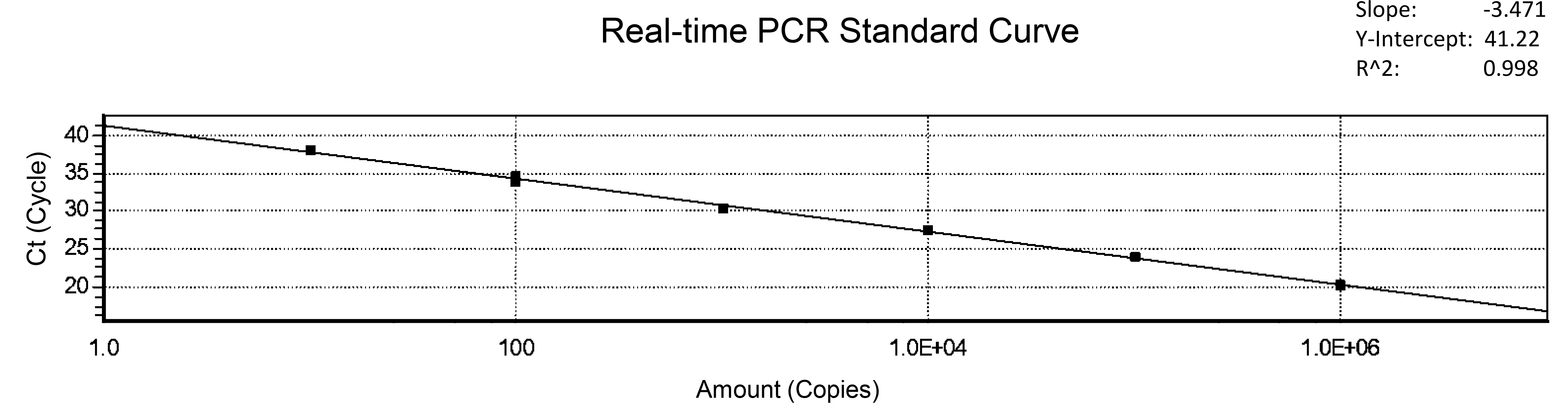

Supplement: Supplementary file 3 — Additional file 3: Figure S1. Real-time PCR standard curve. [file 13071_2023_5881_MOESM3_ESM.tif]

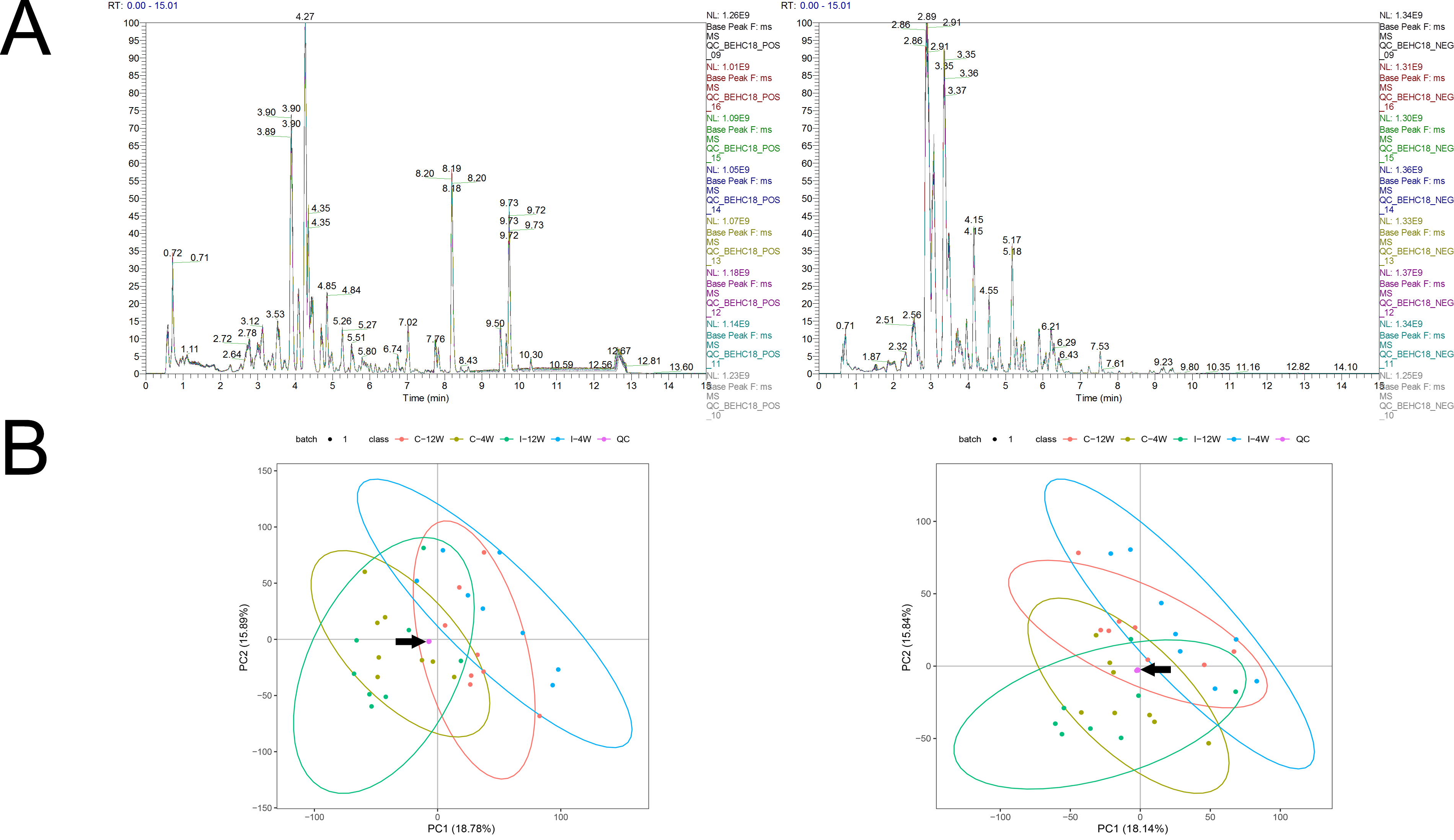

Supplement: Supplementary file 4 — Additional file 4: Figure S2. TIC curves and PCA plots of QC samples. (A) TIC curves of QC samples were highly overlapped. The left represents the ESI + mode, and the right represents the ESI− mode. (B) PCA plots of QC samples. Black arrows point to the points of QC samples that were tightly aggregated. [file 13071_2023_5881_MOESM4_ESM.tif]

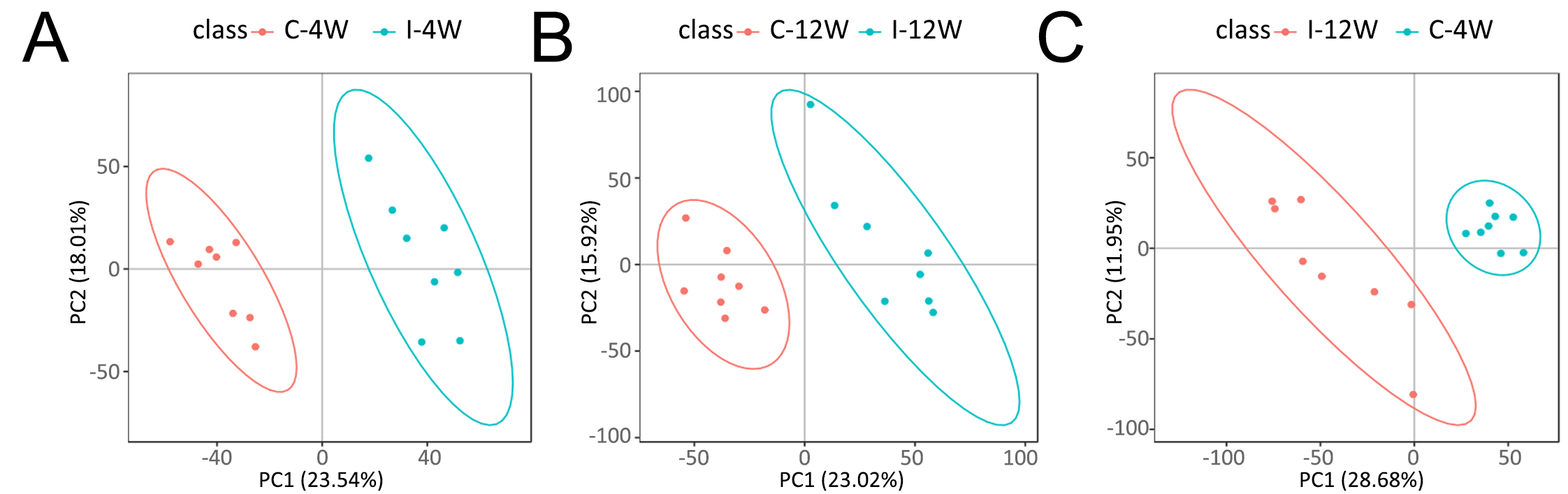

Supplement: Supplementary file 5 — Additional file 5: Figure S3. PLS-DA plots of experimental samples in ESI− mode. (A) Control vs. infection at 4 WPI. R2 = 1.00, Q2 = 0.78. (B) Control vs. infection at 12 WPI. R2 = 0.99, Q2 = 0.62. (C) 4 WPI vs. 12 WPI of the infection group. R2 = 0.99, Q2 = 0.68. [file 13071_2023_5881_MOESM5_ESM.tif]

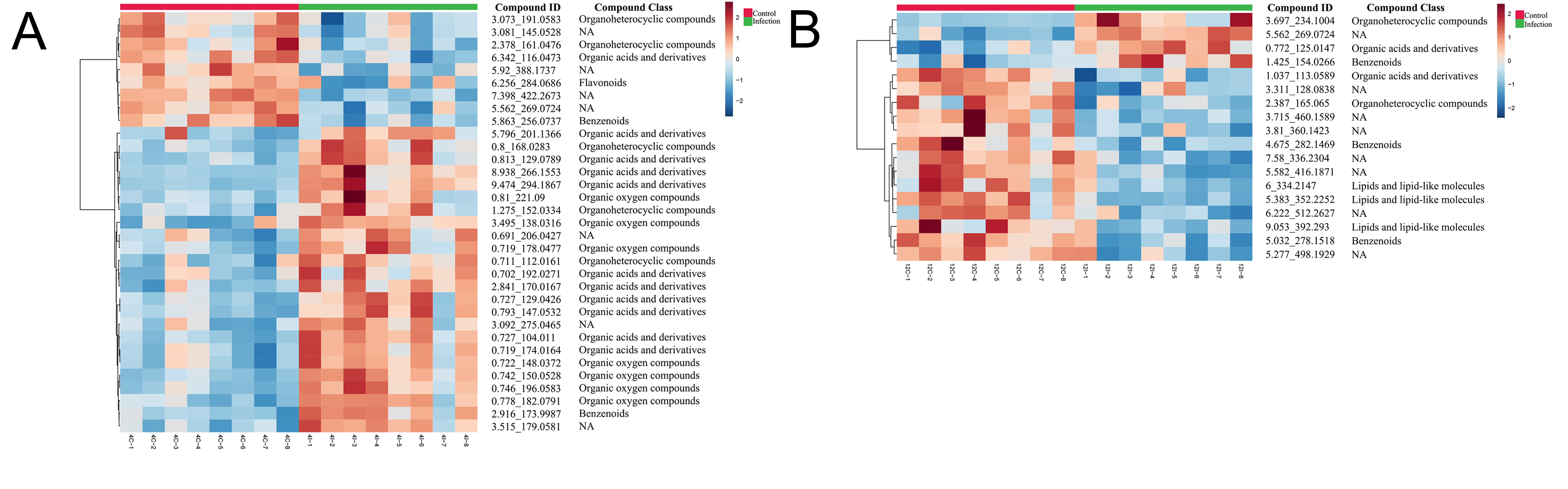

Supplement: Supplementary file 7 — Additional file 7: Heatmaps of differential metabolites at 4 WPI and 12 WPI in ESI− mode. (A) Results at 4 WPI in ESI− mode. (B) Results at 12 WPI in ESI− mode. [file 13071_2023_5881_MOESM7_ESM.tif]
